# 1,4-Bis(2,2′:6′,2′′-terpyridin-4′-yl)benzene

**DOI:** 10.1107/S1600536810047598

**Published:** 2010-11-20

**Authors:** José A. Fernandes, Filipe A. Almeida Paz, Patrícia P. Lima, Severino Alves Jr, Luís D. Carlos

**Affiliations:** a Department of Chemistry, University of Aveiro, CICECO, 3810-193 Aveiro, Portugal; b Department of Physics, University of Aveiro, CICECO, 3810-193 Aveiro, Portugal; c Departamento de Química Fundamental, UFPE, 50590-470, Recife, PE, Brazil

## Abstract

The asymmetric unit of the title compound, C_36_H_24_N_6_, comprises a whole mol­ecule. Supra­molecular inter­actions between neighbouring mol­ecules are essentially π–π stacking inter­actions with small inter­planar distances [3.5140 (15) and 3.6041 (15) Å]. The central phenyl­ene ring is tilted with respect to the two pyridine substituents, subtending angles of 36.17 (11) and 34.95 (11)°. Three of the peripheral pyridine substituents are almost coplanar with the central pyridines [dihedral angles = 5.10 (12)-8.21 (12)°], but one subtends an angle of 24.86 (12)°.

## Related literature

For coordination polymers having the title compound as a bridging ligand, see: Jones *et al.* (2010 ▶); Koo *et al.* (2003 ▶). For oligomeric coordination compounds having the title compound as bridging ligand, see: Maekawa *et al.* (2004 ▶); Schmittel *et al.* (2005 ▶, 2006 ▶). For a description of the Cambridge Structural Database, see: Allen (2002 ▶). For related work from our research group showing the motivation to use aromatic ligands for the design of photoluminescent materials, see: Girginova *et al.* (2007 ▶); Lima *et al.* (2006 ▶, 2009 ▶); Shi *et al.* (2008 ▶). For absolute structure, see: Flack (1983 ▶).
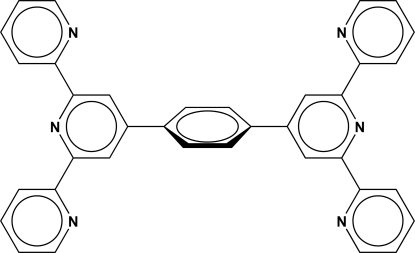


## Experimental

### 

#### Crystal data

C_36_H_24_N_6_
*M* *_r_* = 540.61
Orthorhombic, 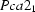

*a* = 9.8493 (2) Å
*b* = 10.0626 (2) Å
*c* = 26.0488 (4) Å
*V* = 2581.69 (8) Å^3^
*Z* = 4
Mo *K*α radiation
μ = 0.09 mm^−1^
*T* = 100 K
0.30 × 0.14 × 0.10 mm

#### Data collection

Bruker X8 Kappa CCD APEXII diffractometer
Absorption correction: multi-scan (*SADABS*; Sheldrick, 1998 ▶) *T*_min_ = 0.975, *T*_max_ = 0.992
39767 measured reflections
3543 independent reflections
2856 reflections with *I* > 2σ(*I*)
*R*_int_ = 0.051

#### Refinement

*R*[*F* ^2^ > 2σ(*F* ^2^)] = 0.042
*wR*(*F* ^2^) = 0.108
*S* = 1.03
3543 reflections
379 parameters
1 restraint
H-atom parameters constrained
Δρ_max_ = 0.38 e Å^−3^
Δρ_min_ = −0.24 e Å^−3^

### 

Data collection: *APEX2* (Bruker, 2006 ▶); cell refinement: *SAINT-Plus* (Bruker, 2005 ▶); data reduction: *SAINT-Plus*; program(s) used to solve structure: *SHELXTL* (Sheldrick, 2008 ▶); program(s) used to refine structure: *SHELXTL*; molecular graphics: *DIAMOND* (Brandenburg, 2009 ▶); software used to prepare material for publication: *SHELXTL*.

## Supplementary Material

Crystal structure: contains datablocks global, I. DOI: 10.1107/S1600536810047598/sj5055sup1.cif

Structure factors: contains datablocks I. DOI: 10.1107/S1600536810047598/sj5055Isup2.hkl

Additional supplementary materials:  crystallographic information; 3D view; checkCIF report

## Figures and Tables

**Fig. 1.**
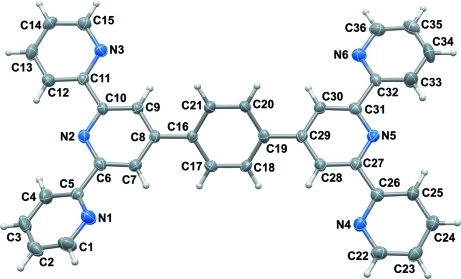
Asymmetric unit of the title compound showing all non-hydrogen atoms represented as thermal ellipsoids drawn at the 80% probability level and hydrogen atoms as small spheres with arbitrary radius.

**Fig. 2.**
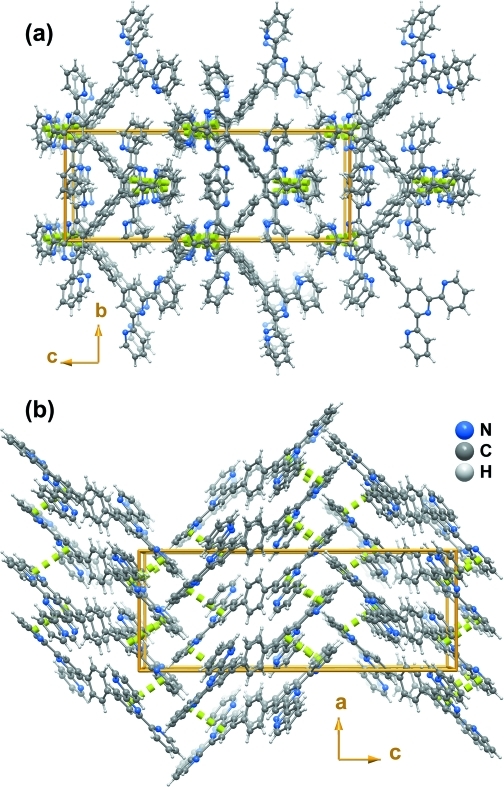
Crystal packing of the title compound viewed in perspective along the (a) [100] and (b) [010] directions of the unit cell. Strong π-π interactions are represented as dashed green lines.

## supplementary crystallographic information

### Comment

The title compound (C_36_H_24_N_6_, see Scheme) can be employed as a ligand in supramolecular chemistry given its hexahapticity and ability to form intermetallic bridges. A survey in the Cambridge Structural Database (Allen, 2002) revealed, however, that only five crystal structures in which this molecule is used as a ligand are known.  While some of these compounds correspond to discrete complexes, namely two dinuclear complexes of zinc and iridium (Schmittel *et al.*, 2006; Maekawa *et al.*, 2004) and a tetranuclear complex of zinc (Schmittel *et al.*, 2005), other two correspond to coordination polymers: a one-dimensional polymer of copper and a three-dimensional framework containing copper and molybdenum (Jones *et al.*, 2010; Koo *et al.*, 2003). Following our interest in the design and synthesis of novel lanthanide photoluminescent complexes (Lima *et al.*, 2006; Lima *et al.*, 2009) and coordination polymers (Shi *et al.*, 2008; Girginova *et al.*, 2007), we isolated crystals of the title compound as a by-product of hydrothermal synthesis.

Despite of the various possible molecular symmetry elements, the asymmetric unit comprises one whole molecule of the title compound (Figure 1). Both terpyridine (terpy) substituents have the three N-atoms in mutual *trans* conformation. The central benzene ring forms angles of 36.17 (11) and 34.95 (11)° with the two neighbouring pyridine (py) substituents, which are almost coplanar [angle between rings 1.22 (11)°]. The terminal py rings subtend small angles with each central py: while three of these dihedral angles are in the 5.10 (12)-8.21 (12)° range, the fourth is slightly larger and measured as 24.86 (12)°. The crystal packing (Figure 2) is mainly driven by the need to effectively fill the available space (van der Waals contacts) in conjunction with a couple of strong π-π stacking interactions. The latter interactions occur between the central py rings and two terminal ones of neighbouring molecular units: distance between centroids of 3.5140 (15) and 3.6041 (15) Å. These interactions (green dashed lines in Figure 2) promote the formation of a two-dimensional supramolecular layer in the *ab* plane.

### Experimental

All chemicals have been purchased from commercial sources and were used as received without any further purification: Eu_2_O_3_ (Sigma-Aldrich, 99.99% purity) and 4',4''''-(1,4-phenylene)bis(2,2':6',2''-terpyridine) (pbt, Sigma-Aldrich, 96% purity, C_36_H_24_N_6_).

A mixture containing pbt (*ca* 0.4 mmol, 0.2162 g) and Eu_2_O_3_ (*ca* 0.1 mmol, 0.0352 g) in *ca* 4 ml of distilled water was stirred at ambient temperature for 5 minutes. The suspension was then transferred to a 8 ml teflon-lined stainless reaction vessel. The reaction took place at 180 °C for approximately 48 h. Large yellow crystals of unreacted pbt were directly isolated from the contents of the vessel, and were washed with copious amounts of water and ethanol before drying under vacuum.

### Refinement

Hydrogen atoms bound to aromatic carbon atoms were located at their idealized positions and were included in the final structural model in riding-motion approximation with C—H = 0.95 Å. The isotropic thermal displacement parameters for these atoms were fixed at 1.2 times *U*_eq_ of the respective parent carbon atom.

A total of 3381 estimated Friedel pairs have been merged and were not used as independent data for the structure refinement. Prior to this strategy, the Flack parameter (Flack, 1983) converged to 0.0 (2).

### Crystal data

**Table:**

C_36_H_24_N_6_	*F*(000) = 1128
*M**_r_* = 540.61	*D*_x_ = 1.391 Mg m^−^^3^
Orthorhombic, *P**c**a*2_1_	Mo *K*α radiation, λ = 0.71073 Å
Hall symbol: P 2c -2ac	Cell parameters from 7316 reflections
*a* = 9.8493 (2) Å	θ = 2.6–30.5°
*b* = 10.0626 (2) Å	µ = 0.09 mm^−^^1^
*c* = 26.0488 (4) Å	*T* = 100 K
*V* = 2581.69 (8) Å^3^	Needle, yellow
*Z* = 4	0.30 × 0.14 × 0.10 mm

### Data collection

**Table:**

Bruker X8 Kappa CCD APEXII diffractometer	3543 independent reflections
Radiation source: fine-focus sealed tube	2856 reflections with *I* > 2σ(*I*)
graphite	*R*_int_ = 0.051
ω and φ scans	θ_max_ = 29.1°, θ_min_ = 3.7°
Absorption correction: multi-scan (*SADABS*; Sheldrick, 1998)	*h* = −13→13
*T*_min_ = 0.975, *T*_max_ = 0.992	*k* = −12→13
39767 measured reflections	*l* = −35→35

### Refinement

**Table:**

Refinement on *F*^2^	Primary atom site location: structure-invariant direct methods
Least-squares matrix: full	Secondary atom site location: difference Fourier map
*R*[*F*^2^ > 2σ(*F*^2^)] = 0.042	Hydrogen site location: inferred from neighbouring sites
*wR*(*F*^2^) = 0.108	H-atom parameters constrained
*S* = 1.03	*w* = 1/[σ^2^(*F*_o_^2^) + (0.0619*P*)^2^ + 0.5147*P*] where *P* = (*F*_o_^2^ + 2*F*_c_^2^)/3
3543 reflections	(Δ/σ)_max_ = 0.001
379 parameters	Δρ_max_ = 0.38 e Å^−^^3^
1 restraint	Δρ_min_ = −0.24 e Å^−^^3^

### Special details

**Table:**

Geometry. All e.s.d.'s (except the e.s.d. in the dihedral angle between two l.s. planes) are estimated using the full covariance matrix. The cell e.s.d.'s are taken into account individually in the estimation of e.s.d.'s in distances, angles and torsion angles; correlations between e.s.d.'s in cell parameters are only used when they are defined by crystal symmetry. An approximate (isotropic) treatment of cell e.s.d.'s is used for estimating e.s.d.'s involving l.s. planes.
Refinement. Refinement of *F*^2^ against ALL reflections. The weighted *R*-factor *wR* and goodness of fit *S* are based on *F*^2^, conventional *R*-factors *R* are based on *F*, with *F* set to zero for negative *F*^2^. The threshold expression of *F*^2^ > σ(*F*^2^) is used only for calculating *R*-factors(gt) *etc*. and is not relevant to the choice of reflections for refinement. *R*-factors based on *F*^2^ are statistically about twice as large as those based on *F*, and *R*- factors based on ALL data will be even larger.

### Fractional atomic coordinates and isotropic or equivalent isotropic displacement parameters (Å^2^)

**Table:**

	*x*	*y*	*z*	*U*_iso_*/*U*_eq_	
N1	0.0080 (2)	0.1240 (2)	1.05763 (10)	0.0216 (5)	
N2	0.1785 (2)	−0.1486 (2)	0.99796 (8)	0.0138 (4)	
N3	0.4382 (2)	−0.3393 (2)	0.93357 (9)	0.0161 (5)	
N4	0.3246 (2)	0.8440 (2)	0.79223 (8)	0.0161 (4)	
N5	0.58036 (19)	0.6498 (2)	0.72756 (8)	0.0139 (4)	
N6	0.7630 (2)	0.3705 (2)	0.67575 (9)	0.0185 (5)	
C1	−0.0869 (3)	0.1423 (3)	1.09389 (12)	0.0254 (6)	
H1	−0.0994	0.2298	1.1068	0.030*	
C2	−0.1674 (3)	0.0429 (3)	1.11374 (11)	0.0247 (7)	
H2	−0.2318	0.0612	1.1400	0.030*	
C3	−0.1519 (3)	−0.0839 (3)	1.09445 (11)	0.0249 (6)	
H3	−0.2074	−0.1545	1.1065	0.030*	
C4	−0.0542 (3)	−0.1068 (3)	1.05716 (11)	0.0196 (6)	
H4	−0.0405	−0.1936	1.0437	0.024*	
C5	0.0235 (3)	−0.0007 (3)	1.03974 (11)	0.0135 (6)	
C6	0.1305 (2)	−0.0238 (3)	1.00011 (10)	0.0139 (5)	
C7	0.1762 (2)	0.0775 (3)	0.96819 (9)	0.0130 (5)	
H7	0.1399	0.1647	0.9711	0.016*	
C8	0.2768 (2)	0.0490 (2)	0.93152 (10)	0.0124 (5)	
C9	0.3273 (2)	−0.0802 (3)	0.92965 (10)	0.0130 (5)	
H9	0.3951	−0.1033	0.9053	0.016*	
C10	0.2773 (2)	−0.1756 (2)	0.96387 (9)	0.0120 (5)	
C11	0.3327 (2)	−0.3128 (3)	0.96507 (10)	0.0131 (5)	
C12	0.2785 (2)	−0.4083 (3)	0.99789 (11)	0.0171 (5)	
H12	0.2052	−0.3865	1.0200	0.020*	
C13	0.3326 (3)	−0.5349 (3)	0.99789 (11)	0.0194 (6)	
H13	0.2958	−0.6019	1.0195	0.023*	
C14	0.4407 (3)	−0.5626 (3)	0.96601 (10)	0.0173 (5)	
H14	0.4802	−0.6487	0.9653	0.021*	
C15	0.4901 (3)	−0.4621 (3)	0.93501 (10)	0.0174 (5)	
H15	0.5654	−0.4813	0.9135	0.021*	
C16	0.3286 (2)	0.1535 (2)	0.89638 (10)	0.0114 (5)	
C17	0.2428 (2)	0.2517 (2)	0.87710 (10)	0.0136 (5)	
H17	0.1497	0.2521	0.8866	0.016*	
C18	0.2921 (2)	0.3487 (2)	0.84423 (9)	0.0131 (5)	
H18	0.2322	0.4149	0.8314	0.016*	
C19	0.4290 (2)	0.3503 (2)	0.82959 (10)	0.0129 (5)	
C20	0.5147 (2)	0.2510 (2)	0.84861 (9)	0.0134 (5)	
H20	0.6076	0.2498	0.8389	0.016*	
C21	0.4653 (2)	0.1544 (2)	0.88147 (10)	0.0138 (5)	
H21	0.5249	0.0878	0.8941	0.017*	
C22	0.2717 (3)	0.9666 (3)	0.78992 (11)	0.0190 (5)	
H22	0.1974	0.9866	0.8119	0.023*	
C23	0.3183 (3)	1.0664 (3)	0.75761 (10)	0.0182 (5)	
H23	0.2777	1.1520	0.7576	0.022*	
C24	0.4259 (2)	1.0367 (3)	0.72547 (11)	0.0177 (5)	
H24	0.4613	1.1024	0.7029	0.021*	
C25	0.4817 (2)	0.9096 (3)	0.72653 (10)	0.0167 (5)	
H25	0.5546	0.8867	0.7043	0.020*	
C26	0.4289 (2)	0.8168 (3)	0.76062 (9)	0.0140 (5)	
C27	0.4838 (2)	0.6781 (3)	0.76221 (10)	0.0128 (5)	
C28	0.4334 (2)	0.5846 (3)	0.79641 (10)	0.0135 (5)	
H28	0.3671	0.6093	0.8211	0.016*	
C29	0.4810 (2)	0.4537 (3)	0.79421 (9)	0.0126 (5)	
C30	0.5797 (2)	0.4234 (2)	0.75747 (9)	0.0130 (5)	
H30	0.6146	0.3358	0.7546	0.016*	
C31	0.6260 (2)	0.5240 (3)	0.72519 (10)	0.0128 (5)	
C32	0.7287 (3)	0.4973 (2)	0.68450 (11)	0.0124 (6)	
C33	0.7851 (3)	0.6012 (3)	0.65635 (11)	0.0227 (6)	
H33	0.7599	0.6905	0.6634	0.027*	
C34	0.8783 (3)	0.5732 (3)	0.61796 (11)	0.0245 (6)	
H34	0.9148	0.6424	0.5973	0.029*	
C35	0.9166 (3)	0.4438 (3)	0.61035 (11)	0.0197 (6)	
H35	0.9834	0.4218	0.5854	0.024*	
C36	0.8562 (3)	0.3464 (3)	0.63969 (10)	0.0203 (6)	
H36	0.8824	0.2568	0.6339	0.024*	

### Atomic displacement parameters (Å^2^)

**Table:**

	*U*^11^	*U*^22^	*U*^33^	*U*^12^	*U*^13^	*U*^23^
N1	0.0219 (10)	0.0193 (12)	0.0237 (12)	0.0009 (9)	0.0076 (10)	−0.0004 (10)
N2	0.0137 (9)	0.0148 (11)	0.0130 (10)	−0.0008 (8)	0.0002 (8)	0.0022 (9)
N3	0.0167 (9)	0.0138 (11)	0.0178 (11)	0.0005 (8)	0.0022 (9)	0.0002 (9)
N4	0.0184 (10)	0.0131 (11)	0.0166 (11)	−0.0005 (8)	0.0013 (9)	0.0006 (9)
N5	0.0130 (9)	0.0134 (10)	0.0154 (10)	0.0005 (8)	−0.0012 (8)	0.0027 (9)
N6	0.0184 (10)	0.0171 (12)	0.0200 (11)	0.0015 (9)	0.0027 (9)	−0.0011 (9)
C1	0.0237 (13)	0.0263 (16)	0.0263 (15)	0.0038 (12)	0.0087 (12)	−0.0051 (12)
C2	0.0182 (12)	0.0396 (19)	0.0164 (13)	0.0049 (13)	0.0051 (11)	0.0043 (14)
C3	0.0190 (13)	0.0325 (17)	0.0230 (14)	−0.0020 (11)	0.0061 (11)	0.0105 (13)
C4	0.0188 (11)	0.0185 (13)	0.0216 (13)	−0.0015 (11)	0.0017 (11)	0.0036 (11)
C5	0.0121 (12)	0.0165 (16)	0.0118 (14)	0.0021 (9)	−0.0024 (11)	0.0021 (9)
C6	0.0112 (10)	0.0178 (12)	0.0127 (12)	−0.0022 (10)	−0.0005 (10)	0.0008 (11)
C7	0.0131 (10)	0.0124 (12)	0.0136 (11)	0.0006 (9)	−0.0011 (9)	0.0000 (10)
C8	0.0121 (11)	0.0131 (14)	0.0121 (12)	−0.0015 (10)	−0.0013 (9)	0.0021 (10)
C9	0.0122 (10)	0.0125 (12)	0.0143 (12)	−0.0010 (9)	0.0002 (9)	0.0013 (10)
C10	0.0128 (10)	0.0125 (12)	0.0107 (11)	−0.0009 (9)	−0.0001 (9)	0.0010 (9)
C11	0.0112 (10)	0.0134 (12)	0.0148 (11)	−0.0020 (9)	−0.0014 (9)	0.0027 (10)
C12	0.0151 (11)	0.0176 (14)	0.0185 (12)	0.0008 (10)	0.0025 (10)	0.0029 (12)
C13	0.0228 (13)	0.0142 (13)	0.0211 (14)	−0.0033 (12)	−0.0005 (11)	0.0049 (12)
C14	0.0197 (12)	0.0117 (13)	0.0206 (13)	0.0021 (10)	−0.0032 (10)	0.0002 (11)
C15	0.0177 (11)	0.0174 (14)	0.0172 (13)	0.0014 (11)	0.0013 (10)	−0.0011 (12)
C16	0.0126 (10)	0.0097 (12)	0.0120 (11)	−0.0008 (9)	0.0009 (9)	0.0005 (9)
C17	0.0109 (10)	0.0152 (12)	0.0147 (11)	−0.0007 (9)	0.0005 (9)	−0.0007 (10)
C18	0.0137 (10)	0.0116 (12)	0.0140 (12)	0.0017 (9)	−0.0015 (9)	0.0021 (10)
C19	0.0153 (11)	0.0128 (12)	0.0105 (11)	−0.0019 (9)	0.0013 (9)	−0.0002 (10)
C20	0.0113 (10)	0.0135 (12)	0.0153 (11)	−0.0004 (9)	0.0032 (9)	0.0008 (9)
C21	0.0141 (10)	0.0117 (12)	0.0156 (12)	0.0008 (9)	0.0002 (10)	0.0016 (10)
C22	0.0183 (12)	0.0172 (13)	0.0213 (14)	0.0023 (11)	0.0014 (11)	−0.0030 (12)
C23	0.0207 (12)	0.0121 (13)	0.0217 (14)	0.0006 (10)	−0.0018 (11)	0.0002 (11)
C24	0.0199 (12)	0.0156 (13)	0.0175 (13)	−0.0006 (11)	−0.0015 (11)	0.0034 (12)
C25	0.0179 (12)	0.0132 (13)	0.0189 (13)	−0.0010 (10)	0.0020 (10)	0.0025 (11)
C26	0.0139 (10)	0.0140 (12)	0.0140 (12)	−0.0011 (9)	−0.0019 (9)	−0.0007 (10)
C27	0.0112 (10)	0.0130 (12)	0.0140 (11)	−0.0015 (9)	−0.0012 (9)	0.0015 (10)
C28	0.0121 (10)	0.0152 (13)	0.0132 (12)	−0.0012 (9)	0.0007 (9)	0.0026 (10)
C29	0.0127 (11)	0.0131 (13)	0.0122 (12)	−0.0004 (10)	−0.0013 (9)	0.0022 (10)
C30	0.0140 (11)	0.0107 (12)	0.0143 (12)	−0.0004 (9)	−0.0002 (10)	0.0006 (10)
C31	0.0105 (10)	0.0164 (12)	0.0117 (12)	−0.0006 (10)	−0.0019 (10)	−0.0003 (11)
C32	0.0108 (11)	0.0164 (16)	0.0101 (14)	−0.0022 (9)	−0.0015 (11)	0.0014 (9)
C33	0.0267 (13)	0.0159 (14)	0.0254 (15)	0.0009 (11)	0.0075 (12)	0.0010 (11)
C34	0.0254 (13)	0.0274 (16)	0.0206 (14)	−0.0090 (12)	0.0057 (11)	0.0070 (12)
C35	0.0160 (11)	0.0290 (16)	0.0139 (12)	0.0010 (11)	0.0012 (10)	−0.0029 (12)
C36	0.0181 (12)	0.0209 (14)	0.0218 (14)	0.0027 (11)	0.0023 (11)	−0.0020 (11)

### Geometric parameters (Å, °)

**Table:**

N1—C1	1.341 (4)	C15—H15	0.9500
N1—C5	1.347 (3)	C16—C17	1.393 (3)
N2—C6	1.342 (3)	C16—C21	1.402 (3)
N2—C10	1.345 (3)	C17—C18	1.386 (3)
N3—C15	1.337 (3)	C17—H17	0.9500
N3—C11	1.351 (3)	C18—C19	1.402 (3)
N4—C22	1.341 (3)	C18—H18	0.9500
N4—C26	1.344 (3)	C19—C20	1.399 (3)
N5—C27	1.342 (3)	C19—C29	1.482 (3)
N5—C31	1.344 (3)	C20—C21	1.383 (3)
N6—C36	1.335 (3)	C20—H20	0.9500
N6—C32	1.340 (3)	C21—H21	0.9500
C1—C2	1.377 (4)	C22—C23	1.388 (4)
C1—H1	0.9500	C22—H22	0.9500
C2—C3	1.380 (5)	C23—C24	1.383 (4)
C2—H2	0.9500	C23—H23	0.9500
C3—C4	1.387 (4)	C24—C25	1.393 (4)
C3—H3	0.9500	C24—H24	0.9500
C4—C5	1.389 (4)	C25—C26	1.390 (4)
C4—H4	0.9500	C25—H25	0.9500
C5—C6	1.493 (4)	C26—C27	1.497 (3)
C6—C7	1.391 (4)	C27—C28	1.388 (3)
C7—C8	1.406 (3)	C28—C29	1.399 (4)
C7—H7	0.9500	C28—H28	0.9500
C8—C9	1.392 (3)	C29—C30	1.397 (3)
C8—C16	1.485 (3)	C30—C31	1.393 (4)
C9—C10	1.400 (3)	C30—H30	0.9500
C9—H9	0.9500	C31—C32	1.490 (4)
C10—C11	1.484 (3)	C32—C33	1.393 (4)
C11—C12	1.392 (4)	C33—C34	1.386 (4)
C12—C13	1.381 (4)	C33—H33	0.9500
C12—H12	0.9500	C34—C35	1.370 (4)
C13—C14	1.379 (4)	C34—H34	0.9500
C13—H13	0.9500	C35—C36	1.378 (4)
C14—C15	1.383 (4)	C35—H35	0.9500
C14—H14	0.9500	C36—H36	0.9500
			
C1—N1—C5	116.7 (2)	C17—C18—C19	120.9 (2)
C6—N2—C10	118.1 (2)	C17—C18—H18	119.6
C15—N3—C11	117.4 (2)	C19—C18—H18	119.6
C22—N4—C26	117.2 (2)	C20—C19—C18	118.4 (2)
C27—N5—C31	117.9 (2)	C20—C19—C29	120.9 (2)
C36—N6—C32	117.8 (2)	C18—C19—C29	120.7 (2)
N1—C1—C2	124.5 (3)	C21—C20—C19	120.6 (2)
N1—C1—H1	117.8	C21—C20—H20	119.7
C2—C1—H1	117.8	C19—C20—H20	119.7
C1—C2—C3	118.1 (3)	C20—C21—C16	120.9 (2)
C1—C2—H2	120.9	C20—C21—H21	119.5
C3—C2—H2	120.9	C16—C21—H21	119.5
C2—C3—C4	119.0 (3)	N4—C22—C23	124.3 (2)
C2—C3—H3	120.5	N4—C22—H22	117.8
C4—C3—H3	120.5	C23—C22—H22	117.8
C3—C4—C5	118.9 (3)	C24—C23—C22	117.7 (3)
C3—C4—H4	120.5	C24—C23—H23	121.1
C5—C4—H4	120.5	C22—C23—H23	121.1
N1—C5—C4	122.7 (2)	C23—C24—C25	119.2 (3)
N1—C5—C6	117.6 (2)	C23—C24—H24	120.4
C4—C5—C6	119.6 (2)	C25—C24—H24	120.4
N2—C6—C7	123.1 (2)	C26—C25—C24	118.8 (2)
N2—C6—C5	115.0 (2)	C26—C25—H25	120.6
C7—C6—C5	121.8 (2)	C24—C25—H25	120.6
C6—C7—C8	119.0 (2)	N4—C26—C25	122.7 (2)
C6—C7—H7	120.5	N4—C26—C27	116.7 (2)
C8—C7—H7	120.5	C25—C26—C27	120.6 (2)
C9—C8—C7	117.8 (2)	N5—C27—C28	122.8 (2)
C9—C8—C16	121.1 (2)	N5—C27—C26	115.8 (2)
C7—C8—C16	121.1 (2)	C28—C27—C26	121.4 (2)
C8—C9—C10	119.5 (2)	C27—C28—C29	119.5 (2)
C8—C9—H9	120.2	C27—C28—H28	120.3
C10—C9—H9	120.2	C29—C28—H28	120.3
N2—C10—C9	122.4 (2)	C30—C29—C28	117.8 (2)
N2—C10—C11	116.1 (2)	C30—C29—C19	120.9 (2)
C9—C10—C11	121.5 (2)	C28—C29—C19	121.3 (2)
N3—C11—C12	122.1 (2)	C31—C30—C29	118.9 (2)
N3—C11—C10	117.0 (2)	C31—C30—H30	120.6
C12—C11—C10	120.9 (2)	C29—C30—H30	120.6
C13—C12—C11	119.3 (2)	N5—C31—C30	123.2 (2)
C13—C12—H12	120.4	N5—C31—C32	115.4 (2)
C11—C12—H12	120.4	C30—C31—C32	121.4 (2)
C14—C13—C12	119.0 (3)	N6—C32—C33	121.6 (2)
C14—C13—H13	120.5	N6—C32—C31	117.7 (2)
C12—C13—H13	120.5	C33—C32—C31	120.7 (2)
C13—C14—C15	118.4 (2)	C34—C33—C32	119.5 (3)
C13—C14—H14	120.8	C34—C33—H33	120.3
C15—C14—H14	120.8	C32—C33—H33	120.3
N3—C15—C14	123.9 (2)	C35—C34—C33	118.6 (3)
N3—C15—H15	118.1	C35—C34—H34	120.7
C14—C15—H15	118.1	C33—C34—H34	120.7
C17—C16—C21	118.6 (2)	C34—C35—C36	118.5 (2)
C17—C16—C8	121.1 (2)	C34—C35—H35	120.8
C21—C16—C8	120.4 (2)	C36—C35—H35	120.8
C18—C17—C16	120.6 (2)	N6—C36—C35	123.9 (3)
C18—C17—H17	119.7	N6—C36—H36	118.0
C16—C17—H17	119.7	C35—C36—H36	118.0
			
C5—N1—C1—C2	0.4 (4)	C18—C19—C20—C21	−0.6 (4)
N1—C1—C2—C3	−1.4 (5)	C29—C19—C20—C21	−179.7 (2)
C1—C2—C3—C4	1.7 (4)	C19—C20—C21—C16	0.1 (4)
C2—C3—C4—C5	−1.1 (4)	C17—C16—C21—C20	0.4 (4)
C1—N1—C5—C4	0.3 (4)	C8—C16—C21—C20	179.8 (2)
C1—N1—C5—C6	−178.8 (3)	C26—N4—C22—C23	−0.6 (4)
C3—C4—C5—N1	0.1 (4)	N4—C22—C23—C24	0.4 (4)
C3—C4—C5—C6	179.2 (2)	C22—C23—C24—C25	0.5 (4)
C10—N2—C6—C7	1.3 (4)	C23—C24—C25—C26	−1.1 (4)
C10—N2—C6—C5	−178.6 (2)	C22—N4—C26—C25	−0.1 (4)
N1—C5—C6—N2	155.2 (2)	C22—N4—C26—C27	−177.9 (2)
C4—C5—C6—N2	−23.9 (4)	C24—C25—C26—N4	0.9 (4)
N1—C5—C6—C7	−24.6 (4)	C24—C25—C26—C27	178.6 (2)
C4—C5—C6—C7	156.3 (3)	C31—N5—C27—C28	2.3 (3)
N2—C6—C7—C8	0.4 (4)	C31—N5—C27—C26	−175.5 (2)
C5—C6—C7—C8	−179.7 (2)	N4—C26—C27—N5	174.9 (2)
C6—C7—C8—C9	−0.9 (3)	C25—C26—C27—N5	−3.0 (3)
C6—C7—C8—C16	179.7 (2)	N4—C26—C27—C28	−2.9 (3)
C7—C8—C9—C10	−0.2 (3)	C25—C26—C27—C28	179.2 (2)
C16—C8—C9—C10	179.1 (2)	N5—C27—C28—C29	−2.0 (4)
C6—N2—C10—C9	−2.5 (4)	C26—C27—C28—C29	175.6 (2)
C6—N2—C10—C11	176.5 (2)	C27—C28—C29—C30	0.7 (4)
C8—C9—C10—N2	2.0 (4)	C27—C28—C29—C19	−179.3 (2)
C8—C9—C10—C11	−177.0 (2)	C20—C19—C29—C30	35.9 (4)
C15—N3—C11—C12	−0.2 (4)	C18—C19—C29—C30	−143.2 (2)
C15—N3—C11—C10	178.9 (2)	C20—C19—C29—C28	−144.0 (2)
N2—C10—C11—N3	−175.6 (2)	C18—C19—C29—C28	36.9 (4)
C9—C10—C11—N3	3.4 (3)	C28—C29—C30—C31	0.2 (3)
N2—C10—C11—C12	3.5 (3)	C19—C29—C30—C31	−179.7 (2)
C9—C10—C11—C12	−177.5 (2)	C27—N5—C31—C30	−1.3 (4)
N3—C11—C12—C13	−1.0 (4)	C27—N5—C31—C32	177.4 (2)
C10—C11—C12—C13	179.9 (2)	C29—C30—C31—N5	0.0 (4)
C11—C12—C13—C14	1.2 (4)	C29—C30—C31—C32	−178.6 (2)
C12—C13—C14—C15	−0.4 (4)	C36—N6—C32—C33	1.5 (4)
C11—N3—C15—C14	1.1 (4)	C36—N6—C32—C31	−179.3 (2)
C13—C14—C15—N3	−0.9 (4)	N5—C31—C32—N6	−170.8 (2)
C9—C8—C16—C17	144.8 (2)	C30—C31—C32—N6	8.0 (4)
C7—C8—C16—C17	−35.9 (3)	N5—C31—C32—C33	8.5 (4)
C9—C8—C16—C21	−34.6 (4)	C30—C31—C32—C33	−172.8 (3)
C7—C8—C16—C21	144.7 (2)	N6—C32—C33—C34	0.6 (4)
C21—C16—C17—C18	−0.5 (3)	C31—C32—C33—C34	−178.6 (3)
C8—C16—C17—C18	−179.9 (2)	C32—C33—C34—C35	−2.8 (4)
C16—C17—C18—C19	0.0 (4)	C33—C34—C35—C36	2.9 (4)
C17—C18—C19—C20	0.5 (4)	C32—N6—C36—C35	−1.4 (4)
C17—C18—C19—C29	179.7 (2)	C34—C35—C36—N6	−0.8 (4)
